# Factors Associated With Evolution of the Use of Medical Assistance in Dying: Protocol for a Scoping Review

**DOI:** 10.2196/85963

**Published:** 2026-04-21

**Authors:** Isabelle Marcoux, David Lavoie, Amy Bergeron, Alexandra Beaudin, Simon Lemyre, Valérie Bourgeois-Guérin, Gina Bravo, Maude Hébert, Chantale Simard, Deborah Ummel, Catherine Perron, Marie-Eve Bouthillier

**Affiliations:** 1 Interdisciplinary School of Health Sciences, Faculty of Health Sciences, University of Ottawa, 125 University Private, Ottawa, ON, H1N6N5, Canada, 1 613-562-5700; 2 Department of Family and Emergency Medicine, Faculty of Medicine, Université de Montréal, Montréal, QC, Canada; 3 Health Library, Direction of Libraries, Université de Montréal, Montréal, QC, Canada; 4 Department of Psychology, Université du Québec à Montréal, Montréal, QC, Canada; 5 Department of Community Health Sciences, Faculty of Medicine and Health Sciences, Université de Sherbrooke, Sherbrooke, QC, Canada; 6 Département des sciences infirmières, Université du Québec à Trois-Rivières, Trois-Rivières, QC, Canada; 7 Department of Health Sciences, Nursing Sciences Module, Université du Québec à Chicoutimi, Chicoutimi, QC, Canada; 8 Institute of Psychology, Faculty of Social and Political Sciences, University of Lausanne, Lausanne, Switzerland; 9 École de travail social, Faculté des lettres et sciences humaines, Université de Sherbrooke, Sherbrooke, QC, Canada

**Keywords:** medical assistance in dying, MAiD, euthanasia, assisted suicide, legislation, rates, deaths, practices, requests, influencing factors

## Abstract

**Background:**

As of June 2025, medical assistance in dying (MAiD) is allowed in over 25 jurisdictions across 12 countries, with varying rates of requests and provision. Hypotheses have been suggested to explain these variations, but they are rarely backed up by empirical evidence. As more jurisdictions consider legalizing MAiD, it is important to better understand what factors may explain the evolution of the use of MAiD worldwide with a systematic approach.

**Objective:**

This scoping review aims to map the available evidence regarding the factors associated with the evolution of the use of MAiD in jurisdictions where it is allowed.

**Methods:**

The scoping review will follow the Joanna Briggs Institute methodology and the PRISMA-ScR (Preferred Reporting Items for Systematic Reviews and Meta-Analyses extension for Scoping Reviews) guidelines. Ten electronic databases (including MEDLINE, Embase, CINAHL Complete, and APA PsycInfo) and additional gray literature sources will be searched from inception to the present. This scoping review will consider multiple types of publications (eg, primary studies, research syntheses, and government reports) and will report factors associated with the use of MAiD for people who have requested or died by MAiD in jurisdictions that have allowed it for at least 5 years. Publications in English, French, Spanish, German, or Dutch will be included. Screening for assessment against the inclusion criteria and data extraction will be carried out independently by pairs of reviewers. Findings will be presented in a narrative format and mapped into tables and graphs to address the review aims.

**Results:**

The database search for scientific publications was completed in March 2025. A total of 8570 publications were identified after removing duplicates. As of October 2025, title and abstract screening is complete, with 216 articles retained for the next stage. The full-text review is underway and scheduled to be completed by December 2025, and results are expected to be submitted for publication in 2026.

**Conclusions:**

MAiD is gaining legal and policy attention worldwide, with wide variation in rates of request and provision over time across jurisdictions. This scoping review will contribute to mapping and synthesizing evidence on the factors that may explain these variations.

## Introduction

### Background

In the past decades, there has been a growing movement in various parts of the world to legalize assisted death practices. Twenty years ago, only a few jurisdictions allowed such practices, including the state of Oregon, Belgium, Colombia, the Netherlands, Luxembourg, and Switzerland. However, since 2015, assisted death practices have been authorized in additional US states and in other countries, such as Canada, New Zealand, Spain, Austria, and Portugal, as well as in most Australian states [1]. Assisted death practice legislation across jurisdictions generally shares core eligibility criteria (eg, minimum age and health conditions) and procedural safeguards (eg, physician or authorized person consultations and reporting processes), but there are also variations [2]. Some regulations of assisted death practices are more general and liberal, whereas others are rather specific and restrictive. In addition, some jurisdictions broadened their eligibility criteria after the law came into force to allow more patients to access the procedure. In Canada, for example, the passing of Bill C-7 in 2021 expanded access to some individuals whose death was not “reasonably foreseeable” [3]. As legislative proposals and court decisions on the issue become more frequent in many legislations, policymakers and researchers are questioning why people request and die by assisted death practices [4].

Different terms are used to define assisted death practices depending on the legislations and regulations. “Euthanasia” is defined as the act of intentionally ending the life of a patient by a qualified health care practitioner through medical means at that patient’s explicit request, whereas “physician-assisted suicide” (PAS) refers to the provision or prescription of drugs by a health care practitioner for a patient to end their own life [2]. Some jurisdictions allow only euthanasia, whereas others permit only PAS or both practices. This protocol will use “medical assistance in dying” (MAiD) for both the literature covering euthanasia and PAS and other regulated terms (eg, “medical aid in dying,” “termination of life,” and “voluntary assisted dying”), but specific terms will be used when there are issues related to either of these practices. Therefore, unless otherwise specified, we will use “MAiD” as the overarching term in this review.

Reported numbers of deaths by MAiD vary greatly over time between jurisdictions where such practices are allowed. In Belgium, where the Act on Euthanasia took effect in 2002, the number of reported deaths by euthanasia increased from 0.2% of all deaths in 2003 [5] to 3.1% in 2023 [6]. In comparison, only 6 years after the legalization of MAiD in Canada, the proportion of all deaths attributed to MAiD rose from 0.7% in the first full year of legalization in 2016-2017 (as this proportion was not available at that time, we made our own calculation based on the total number of MAiD cases reported in the first and second interim updates [7,8] and on mortality data drawn from Statistics Canada [9]) to 4.7% of all deaths in 2023 [10]. Furthermore, within the same country, the rates of MAiD provisions vary significantly across states or provinces. For instance, in 2022, Canadian provinces experienced different variations in MAiD provisions: Saskatchewan had the lowest year-over-year increase (4.0%); Quebec, which has its own law regulating MAiD that came into force 1 year before the federal law [11], saw the highest growth rates (45.5%); whereas Manitoba reported a decline (−9.0%). Regional differences are also notable within a given province: in Quebec, the proportion of deaths by MAiD reported by different health regions in 2023 ranged from less than 4% to over 10.5% [12].

Some assumptions have been formulated to explain the increase in MAiD rates in certain jurisdictions. Boer [13] describes the development of the practice of MAiD in the Netherlands from 1968 onward, noting that the number of deaths by euthanasia tripled between 2007 and 2021, a period characterized by the expansion of eligibility criteria and the implementation of mobile teams to improve access to euthanasia for those without a prior physician-patient relationship. Pullman [14] explores factors that may explain Canada’s growing and higher rates of MAiD deaths compared to California’s despite their respective laws taking effect in the same year (2016). He suggests that, in Canada, eligibility criteria are more ambiguous, their interpretation is more flexible, and the method of provision (injection rather than prescription of lethal drugs) is more direct. For others, the high rise in MAiD deaths in Canada may be clinician dependent, suggesting that most practices are performed by a few highly active clinicians [15].

### What This Scoping Review Will Contribute

To date, most of the published work is based on opinions or demonstrations focused on specific explanations of the different rates without aiming to be comprehensive. Some reviews address parts of the issue—for example, they focus on factors influencing requests [16], decisions [17], or access to MAiD [18]; on one specific factor [19,20]; on barriers to and facilitators of engaging in the practice of MAiD [21]; or on challenges related to implementing MAiD from a health system perspective [22]. One systematic review published in 2013 [23] provides valuable background for our topic, although it focuses on individual characteristics (eg, sex, age, marital status, educational level, and condition leading to PAS or euthanasia) associated with numbers of and variations in assisted death practices in different countries where these practices were legal. Thus, other potential associated factors such as organizational, sociocultural, or legal context were not considered.

These reviews show that there are available data on the subject. However, after a preliminary search of MEDLINE, the Cochrane Database of Systematic Reviews, *JBI Evidence Synthesis*, and PROSPERO, we found no reviews (published or planned) with the specific aims to identify factors associated with the evolution of the use of MAiD (as defined below). To address this gap, the proposed scoping review will provide a comprehensive overview of the available evidence regarding these factors. Better knowledge of the available data regarding these factors could inform decision-makers and health care professionals, as well as jurisdictions contemplating the legalization of these practices.

This proposed scoping review is part of a larger research study funded by the government of Quebec (Canada) [24] to explore the rapid rise in MAiD rates in the province. From April 1, 2023, to March 31, 2024, a total of 7.3% of all deaths in Quebec were a result of MAiD, the highest rate in jurisdictions where it is legal [25]. In the call for proposals, the government of Quebec also expressed interest in learning from other jurisdictions where MAiD is allowed. Therefore, to provide a comprehensive picture of the factors influencing the evolution in the use of MAiD from an international comparison perspective, the proposed scoping review aims to map the available evidence from jurisdictions where such practices have been allowed for at least 5 years.

### Review Questions

#### Main Question

The main question addressed by this scoping review is as follows: What factors are associated with the evolution in the use of MAiD in jurisdictions where such practices have been allowed for at least 5 years?

#### Subquestions

The subquestions are as follows: (1) What types of evidence sources are available on factors influencing the evolution in the use of MAiD (eg, primary research, evidence syntheses, and discussion articles)? (2) Who provides the evidence on factors associated with the evolution in the use of MAiD (eg, patients, relatives, MAiD practitioners, governments, or regulatory commissions)? (3) What are the target populations (adults who have requested or died by MAiD) for the available evidence? (4) What factors were identified as influencing the evolution in the use of MAiD?

Findings from this scoping review will also be used to inform a realist review [26], which will explore what are the effects of MAiD legislations and public policies on MAiD practices in different jurisdictions and why they produce such effects, for whom, and in which circumstances.

## Methods

### Study Design and Review Team

The proposed scoping review will be conducted in accordance with the Joanna Briggs Institute (JBI) methodology for scoping reviews [27] and reported in line with the PRISMA-ScR (Preferred Reporting Items for Systematic Reviews and Meta-Analyses extension for Scoping Reviews; Checklist 1) [28]. This protocol has been registered in Open Science Framework [29]. The interdisciplinary review team consists of researchers in psychology (IM, DL, A Beaudin, DU, and VB-G), nursing (CS and MH), mathematics (GB), and clinical ethics (CP, MEB, and SL), as well as a professional librarian with expertise in information retrieval for knowledge synthesis (A Bergeron). Research team members discussed and revised the drafted protocol, bringing together scientific, clinical, and ethical expertise.

It is worth noting that, prior to this scoping review protocol, we considered conducting a mixed methods systematic review (MMSR). However, in an MMSR, nonempirical evidence (eg, expert opinion–based pieces, government reports, and discussion papers) and literature reviews are excluded. While these sources may not fit the MMSR criteria, they could provide valuable information for our research question. Given the aims of our review, a scoping review offers the most suitable framework [30,31].

### Eligibility Criteria

According to the JBI methodology for scoping reviews, the eligibility criteria are based on the population, concept, and context format, as well as the type of sources.

#### Population

The population of interest is adults who have requested or died by MAiD. However, data concerning this phenomenon may be reported by a variety of populations, such as the patients themselves; relatives; substitute decision-makers, surrogates, or proxies; MAiD providers (physicians, nurse practitioners, or other authorized practitioners); or other health care professionals who were involved in the process of MAiD requests, practices, or deaths (eg, social workers, psychologists, or spiritual care advisors). Studies conducted with key informants, such as members of a regulatory organization for MAiD, representatives of a professional body, or any other type of informant (eg, decision-makers or nonclinical leaders), will also be considered for inclusion. Publications that investigate factors related to the use of MAiD through secondary data (eg, analysis based on death statistics and government reports) will also be included.

#### Concept

The use of MAiD is conceptualized as MAiD requests, practices, or deaths. MAiD requests are demands made to any health care professional by a patient and that could lead to the assessment of the patient’s eligibility. Practices include the assessment of a patient for MAiD or the provision of MAiD to an eligible patient. Assessment refers to a clinician completing an evaluation to ensure that the patient meets all eligibility criteria for MAiD in their jurisdiction. Provision of MAiD is the injection or prescription of medications to intentionally end the life of the patient who meets the eligibility criteria at the person’s request. Evolution is conceptualized as the development (change or stability over time) of the use of MAiD.

Factors influencing the evolution in the use of MAiD are defined as a characteristic that can be perceived, observed, measured, or reported. Drawing on the ecological systems model by Bronfenbrenner [32] (from the microsystem to the macrosystem), these factors are identified and categorized into 4 main types: individual characteristics, clinical or organizational factors, sociocultural context, and laws and policies.

Exclusion criteria are publications investigating only passive euthanasia or suicide, focusing solely on opinions or attitudes toward MAiD without an explicit association with the evolution in the use of MAiD, or studying only the factors associated with the use of MAiD (at one point in time) without considering its evolution.

#### Context

The inclusion criterion of time (selecting jurisdictions where MAiD has been legally allowed for at least 5 years) is important to ensure that sufficient data are available to observe meaningful trends and variations over time. As of December 3, 2024 (date of our database search for preliminary pilot selection; Multimedia Appendix 1), 16 jurisdictions were considered for inclusion: Australia (Victoria); Belgium; Canada; Colombia; Luxembourg; the Netherlands; Switzerland; and California, Maine, Montana, New Jersey, Hawaii, Colorado, Oregon, Vermont, Washington, and the District of Columbia in the United States. All types of settings are included (eg, hospitals, long-term care facilities, private dwellings, and hospices).

Existing prelegalization data from these jurisdictions will also be included. For example, in the Netherlands, although the law was passed in 2001 and came into force in 2002, the practices had been regulated by the medical authorities since the early 1990s. Relevant data have been published (prior to legalization [33-35]) and are important to capture.

#### Types of Sources

To provide a thorough understanding of the topic, this review will incorporate all available sources of information on factors influencing the use of MAiD, including both peer-reviewed publications and gray literature. We will consider reports of primary or secondary data sources using any type of design (quantitative, qualitative, or mixed methods), as well as reviews and documents that the JBI describes as “textual evidence” (eg, expert opinion–based pieces, government or institutional policies and/or reports, unpublished [or gray] literature, and discussion papers) [36]. Research protocols, book reviews, media publications, and conference proceedings will be excluded.

### Search Strategy

The primary search strategy for MEDLINE was developed iteratively by one author (A Bergeron), a medical librarian, in collaboration with other authors (DL and IM), as recommended in the *JBI Manual for Evidence Synthesis* [27], and reported according to the PRISMA-S (Preferred Reporting Items for Systematic Reviews and Meta-Analyses extension for reporting literature searches in systematic reviews) guidelines (Checklist 2) [37]. The key concepts for the strategy were identified from an initial set of target articles as well as several test searches: (1) MAiD, (2) requests for or provision of MAiD or statistical data pertaining to cases, and (3) jurisdictions in which MAiD has been allowed for at least 5 years. No restrictions were applied based on publication date. The full search strategies will be reported in the final manuscript according to PRISMA-S guidelines.

Although the evolution in the use of MAiD is central to this scoping review, our preliminary tests revealed that the terminology describing this concept is often heterogeneous and subtle. Furthermore, including this concept in the search strategy sometimes excluded relevant articles. Consequently, it was not retained for the initial search strategy but, rather, serves as an inclusion criterion during the screening process.

The preliminary strategy was revised by the team and additionally reviewed by a second librarian using the PRESS (Peer Review of Electronic Search Strategies) guidelines, as additionally recommended by the JBI [26]. The MEDLINE search strategy was then finalized and has been adapted for other sources using database-appropriate subject headings and platform-specific syntax. In total, 10 electronic databases were searched: MEDLINE (Ovid), Embase (Ovid), CINAHL Complete (EBSCO), APA PsycInfo (Ovid), Web of Science Core Collection, Social Sciences Abstracts (EBSCO), Sociological Abstracts (ProQuest), Dissertations and Theses Closed (ProQuest), and Persée and Érudit (Multimedia Appendix 1). A process is currently being developed to search for gray literature sources for additional documents (eg, government or institutional policies and/or reports and position papers). Subsequently, backward and forward citation tracking will be performed for references in the initial corpus of included articles using Web of Science. References of and articles citing the included articles will be exported to Covidence (Veritas Health Innovation) and screened for additional articles.

### Study and Source of Evidence Selection

Following the execution of the full search strategy, references were exported to Covidence, and duplicates were removed using its automatic detection feature. Following a pilot test of 100 records (conducted independently by DL, IM, A Beaudin, and SL), titles and abstracts were screened by at least 2 independent reviewers (DL, A Beaudin, and SL) for assessment against the inclusion criteria, which has now been completed. Publications in English, French, Spanish, German, or Dutch were considered for inclusion. At each stage (past, current, and next) of the selection process, any disagreements that arise between reviewers are resolved through discussion or by including a third party (IM). The full texts of selected citations are currently being assessed in detail against the inclusion criteria by at least 2 independent reviewers. Reasons for the exclusion of sources of evidence at the full-text stage are recorded and will be reported in full, as will the results of the search and the study inclusion process, in the final scoping review and will be presented in a PRISMA (Preferred Reporting Items for Systematic Reviews and Meta-Analyses) flow diagram.

### Data Extraction

Following selection, data will be extracted directly within Covidence by independent reviewers using a data extraction tool developed by the team (Textbox 1). Data will include author; title; source of publication; purpose of study; type of study; date when data were collected; jurisdiction studied; practices studied (eg, MAiD and PAS); settings; and specific details relevant to the review subquestions, such as (1) the type of evidence, (2) the participants providing the information, (3) the target population studied, and (4) factors influencing the evolution in the use of MAiD, which will be divided into 4 main categories (individual characteristics, clinical or organizational factors, sociocultural context, and laws and policies) in accordance with the ecological systems model by Bronfenbrenner [32]. The extraction tool will be pilot-tested on a minimum of 5 articles and then revised. If necessary, authors of the included articles will be contacted to request further information during data extraction. Data extraction, as well as the analysis and presentation of results, will follow the JBI’s recommendations [38].

Textbox 1. **Preliminary categories for the draft data extraction tool.**

- Authors and year
- Title
- Source of publication (eg, scientific journal or government report)
- Purpose of the publication
- Type of evidence (eg, primary research, evidence synthesis, or discussion article)
- Type of study (quantitative, qualitative, or mixed methods)
- Dates of data collection
- Jurisdictions studied
- Performed practices (eg, medical assistance in dying [MAiD], physician-assisted suicide, euthanasia, or voluntary assisted dying)
- Settings (eg, hospitals, long-term care facilities, private dwellings, hospices, or other health care settings)
- Participants who provide the evidence on the factors (eg, patients, relatives, MAiD practitioners, governments, or regulatory commissions)
- Target population (adults who have requested or died by MAiD)
- Factors influencing the evolution of MAiD (individual characteristics, clinical or organizational factors, sociocultural context, and laws and policies)

### Data Analysis and Presentation

Findings will be presented in a narrative format and mapped into tables and graphs to address the review aims (eg, a world map of included jurisdictions with numbers of related publications and a chart of included types of evidence and types of performed practices). In particular, reported factors influencing the evolution of MAiD (categorized in accordance with our ecological systems model) will be analyzed and presented graphically while taking into consideration the types of use of MAiD (either requests, practices, or deaths), as well as the types of evidence (eg, primary research, evidence syntheses, and discussion articles). However, as mentioned by Peters et al [39], because of the broad and iterative nature of the scoping review, it is likely that potentially new relevant information will emerge from searches, screening, and extraction. Accordingly, the search, extraction forms, and presentation of data may be modified and expanded during the review process. All deviations from the protocol will be reported in the review.

## Results

The project was funded in July 2024. We completed the database search in March 2025 and identified 8570 publications after removing duplicates via Covidence. As of October 2025, the screening of titles and abstracts is complete, resulting in the inclusion of 216 articles for the next stage. The full-text review selection is currently underway and scheduled to be completed by December 2025, followed by the data extraction and analysis phases. The full article is planned for submission to a peer-reviewed journal in 2026.

## Discussion

### Expected Findings

To the best of our knowledge, this will be the first review to map the available evidence concerning the factors associated with the evolution in the use of MAiD considering all jurisdictions where MAiD has been legally allowed for at least 5 years as of December 2024. This scoping review is also part of a larger research project in Quebec, where MAiD accounts for the highest proportion of deaths among jurisdictions where it is allowed. The scoping review will contribute to the project’s overarching objective, which is to explain the rapid increase in MAiD in the province, by providing an overview of associated factors while considering the types and characteristics of the existing evidence. Findings from this scoping review will also inform a realist review on how MAiD laws and policies influence practices, for whom, how, and in what contexts. This will be possible by making comparisons of the included jurisdictions’ particularities, which will include specific aspects regarding their laws, their health care systems, and the social acceptability of MAiD, among other aspects. A better knowledge of these factors can also be helpful for health care practitioners, researchers, and policymakers in assessing complex end-of-life care situations and services in their settings and jurisdictions.

### Limitations

Our study will face limitations related to its focus and broad concepts. First, the decision to include only jurisdictions where MAiD has been allowed for at least 5 years may exclude recent but potentially relevant experiences from other jurisdictions. However, this criterion should enable us to assess trends in the use of MAiD, which requires a significant time frame. Second, factors associated with the use of MAiD but not related to its evolution are excluded, which may limit our understanding of potentially important information less likely to be reported as evolutionary use factors, such as family decision-making. Third, the predetermined conceptualization of factors related to the evolution in the use of MAiD may limit the breadth and depth of analysis as this approach is guided by the ecological systems model by Bronfenbrenner [32] and purposefully encompasses variables at different ecological systems (eg, individual, organizational, societal, and legislative). Conversely, this approach may strengthen the identification and analysis of literature gaps based on the types of studied factors, thereby guiding future research.

### Conclusions

Since the 90s, and more particularly for the past 10 years, MAiD has been increasingly debated, and related legislation has been adopted in different countries in America, Europe, and Oceania. The frequency of requests and provision of MAiD and the evolution over time vary widely between jurisdictions. The synthesis of available evidence on factors explaining these variations will be greatly helpful to inform policymakers, clinicians, researchers, and all those concerned about assisted death, end-of-life care, and palliative care.

## Supplementary material

10.2196/85963 Multimedia Appendix 1 Search strategy for MEDLINE (Ovid).

10.2196/85963 Checklist 1 PRISMA-ScR checklist.

10.2196/85963 Checklist 2 PRISMA-S checklist.

## Abbreviations

JBI: Joanna Briggs Institute
MAiD: medical assistance in dying
MMSR: mixed methods systematic review
PAS: physician-assisted suicide
PRESS: Peer Review of Electronic Search Strategies
PRISMA: Preferred Reporting Items for Systematic Reviews and Meta-Analyses
PRISMA-S: Preferred Reporting Items for Systematic Reviews and Meta-Analyses extension for Reporting Literature Searches in Systematic Reviews
PRISMA-ScR: Preferred Reporting Items for Systematic Reviews and Meta-Analyses extension for Scoping Reviews

## References

[1] Voluntary assisted dying. End of Life Law in Australia.

[2] Mroz S, Dierickx S, Deliens L, Cohen J, Chambaere K (2021). Assisted dying around the world: a status quaestionis. Ann Palliat Med.

[3] (2021). Bill C-7: an Act to amend the Criminal Code (medical assistance in dying). Parliament of Canada.

[4] Downar J, MacDonald S, Buchman S (2023). What drives requests for MAiD?. CMAJ.

[5] Premier rapport aux chambres législatives 22 septembre 2002 - 31 décembre 2003. Commission fédérale de Contrôle et d’Évaluation de l’Euthanasie.

[6] Euthanasie – chiffres de l’année 2023. Commission fédérale de Contrôle et d’Évaluation de l’Euthanasie.

[7] Interim update on medical assistance in dying in Canada June 17 to December 31, 2016. Government of Canada.

[8] (2017). 2nd interim report on medical assistance in dying in Canada. Government of Canada.

[9] (2026). Deaths, by month. Statistics Canada.

[10] (2023). Fifth annual report on medical assistance in dying in Canada 2023. Health Canada.

[11] (2025). s-32.0001 - Act respecting end-of-life care. Légis Québec.

[12] (2023). Fourth annual report on medical assistance in dying in Canada 2022. https://www.canada.ca/content/dam/hc-sc/documents/services/medical-assistance-dying/annual-report-2022/annual-report-2022.pdf.

[13] Boer TA (2022). Assisted dying in the Netherlands: recent legal and religious considerations. Rev Derecho Relig.

[14] Pullman D (2023). Slowing the slide down the slippery slope of medical assistance in dying: mutual learnings for Canada and the US. Am J Bioeth.

[15] Lyon C, Lemmens T, Kim SYH (2025). Canadian medical assistance in dying: provider concentration, policy capture, and need for reform. Am J Bioeth.

[16] Castelli Dransart DA, Lapierre S, Erlangsen A (2021). A systematic review of older adults’ request for or attitude toward euthanasia or assisted-suicide. Aging Ment Health.

[17] Xu H, Stjernswärd S, Glasdam S, Fu C (2024). Circumstances affecting patients’ euthanasia or medically assisted suicide decisions from the perspectives of patients, relatives, and healthcare professionals: a qualitative systematic review. Death Stud.

[18] Hewitt J, Wilson M, Bonner A, Bloomer MJ (2024). Factors that influence access to medical assistance in dying services: an integrative review. Health Expect.

[19] Corcoran E, Bird M, Batchelor R, Ahmed N, Nowland R, Pitman A (2024). The association between social connectedness and euthanasia and assisted suicide and related constructs: systematic review. BMC Public Health.

[20] Rahimian Z, Rahimian L, Lopez-Castroman J (2024). What medical conditions lead to a request for euthanasia? A rapid scoping review. Health Sci Rep.

[21] Légère K, Doucet S, Luke A, Goudreau A (2024). Barriers and facilitators for engaging in the practice of medical assistance in dying among providers in Canada: a scoping review protocol. JBI Evid Synth.

[22] Fujioka JK, Mirza RM, Klinger CA, McDonald LP (2019). Medical assistance in dying: implications for health systems from a scoping review of the literature. J Health Serv Res Policy.

[23] Steck N, Egger M, Maessen M, Reisch T, Zwahlen M (2013). Euthanasia and assisted suicide in selected European countries and US states: systematic literature review. Med Care.

[24] (2024). Quebec funds a project to better understand the use of medical assistance in dying. CityNews Montreal.

[25] (2024). Rapport annuel d’activités: du 1er avril 2023 au 31 mars 2024. Commission Sur les Soins de Fin de Vie.

[26] Pawson R, Greenhalgh T, Harvey G, Walshe K (2005). Realist review--a new method of systematic review designed for complex policy interventions. J Health Serv Res Policy.

[27] Peters MD, Godfrey C, McInerney P, Munn Z, Tricco AC, Khalil H, Aromataris E, Lockwood C, Porritt K, Pilla B, Jordan Z (2024). JBI Manual for Evidence Synthesis.

[28] Tricco AC, Lillie E, Zarin W (2018). PRISMA extension for scoping reviews (PRISMA-ScR): checklist and explanation. Ann Intern Med.

[29] Lavoie D, Marcoux I, Bergeron A, Perron C, Bouthillier ME (2025). Factors associated with medical assistance in dying and its evolution: a scoping review protocol. JMIR Research Protocols.

[30] Munn Z, Peters MDJ, Stern C, Tufanaru C, McArthur A, Aromataris E (2018). Systematic review or scoping review? Guidance for authors when choosing between a systematic or scoping review approach. BMC Med Res Methodol.

[31] Peters MDJ, Godfrey C, McInerney P (2022). Best practice guidance and reporting items for the development of scoping review protocols. JBI Evid Synth.

[32] Bronfenbrenner U (1981). The Ecology of Human Development: Experiments by Nature and Design.

[33] Onwuteaka-Philipsen BD, Brinkman-Stoppelenburg A, Penning C, de Jong-Krul GJF, van Delden JJM, van der Heide A (2012). Trends in end-of-life practices before and after the enactment of the euthanasia law in the Netherlands from 1990 to 2010: a repeated cross-sectional survey. Lancet.

[34] van Alphen JE, Donker GA, Marquet RL (2010). Requests for euthanasia in general practice before and after implementation of the Dutch Euthanasia Act. Br J Gen Pract.

[35] van der Heide A, van Delden JJM, Onwuteaka-Philipsen BD (2017). End-of-life decisions in the Netherlands over 25 years. N Engl J Med.

[36] Pearson A, Jordan Z, McArthur A, Aromataris E, Lockwood C, Porritt K, Pilla B, Jordan Z (2024). JBI Manual for Evidence Synthesis.

[37] Rethlefsen ML, Kirtley S, Waffenschmidt S (2021). PRISMA-S: an extension to the PRISMA statement for reporting literature searches in systematic reviews. Syst Rev.

[38] Pollock D, Peters MDJ, Khalil H (2023). Recommendations for the extraction, analysis, and presentation of results in scoping reviews. JBI Evid Synth.

[39] Peters MDJ, Marnie C, Tricco AC (2020). Updated methodological guidance for the conduct of scoping reviews. JBI Evid Synth.

