# Supplementary material for: Factors Associated With Evolution of the Use of Medical Assistance in Dying: Protocol for a Scoping Review
Source: JMIR Res Protoc. 2026 Apr 21;15:e85963. doi: 10.2196/85963 (PMC13098725; doi:10.2196/85963)
Supplement: Multimedia Appendix 1 [file resprot-v15-e85963-s001.docx]

## **Multimedia Appendix 1**

Search Strategy for Medline (Ovid)

This version of the strategy was reported in Lavoie, Marcoux, Bergeron, Perron & Bouthillier [28], published with a CC-BY license.

Search conducted: December 3, 2024

Ovid MEDLINE(R) ALL <1946 to December 02, 2024>

| **#** | **Search** | **Results** |
| --- | --- | --- |
| 1 | Euthanasia/ or Euthanasia, Active/ or Euthanasia, Active, Voluntary/ | 9924 |
| 2 | Suicide, Assisted/ | 6585 |
| 3 | ((assist* or aid) adj4 (suicid* or death* or dying)).tw,kf,jw. | 6808 |
| 4 | euthanasi*.tw,kf,jw. | 25245 |
| 5 | termination of life.tw,kf. | 204 |
| 6 | ((intentional* or active* or explicit* or medication* or pharma* or medicin* or drug* or medical* or administ*) adj3 (hasten* adj3 (death* or dying))).tw,kf. | 102 |
| 7 | or/1-6 | 33071 |
| 8 | (animal or animals or bitch or canine* or cat or cats or dog or dogs or feline or hamster* or lamb or lambs or mice or monkey or monkeys or macaque or mouse or murine or pig or pigs or piglet* or porcine or swine or primate* or rabbit* or rats or rat or rodent* or sheep* or ovine or horse* or equine or chicken* or broiler chick* or turtle* or cephalopod* or mollusc* or mollusk* or dove or doves or fish or insect* or cockroach* or veterina*).ti,kw,jw. | 3074184 |
| **9** | **7 not 8 [concept 1: MAiD]** | **26457** |
| 10 | exp Statistics as Topic/ | 3180072 |
| 11 | Euthanasia/sn or exp Euthanasia, Active/sn or Suicide, Assisted/sn | 596 |
| 12 | (request* or "ask for" or "asking for" or choos* or choic* or pursuit or pursue$1 or pursuing or decision* or recours*).tw,kf. [demandes] | 1224895 |
| 13 | (practice* or participat* or refus* or provid* or provision* or induce$1 or inducing or administ* or inject*).tw,kf. [administration] | 10126201 |
| 14 | (case* or statistic* or number* or epidemiolog* or public data or official pronounc* or official record* or deaths or suicides).tw,kf. [taux & données numériques] | 8243927 |
| **15** | **10 or 11 or 12 or 13 or 14 [concept 2: requests, administration, case statistics]** | **17729764** |
| 16 | exp Canada/ or (canada or canadian or alberta or british columbia or manitoba or new brunswick or newfoundland or labrador or northwest territories or nova scotia or nunavut or ontario or prince edward island or quebec or saskatchewan or yukon or montreal or sherbrooke or toronto or hamilton or ottawa or victoria or vancouver).tw,kf,jw. | 542711 |
| 17 | Netherlands/ or (netherlands or amsterdam or utrecht or leiden or nijmegen).tw,kf,jw. | 126834 |
| 18 | Belgium/ or (belgium or belge or leuven or ghent or brussels).tw,kf,jw. | 48357 |
| 19 | Switzerland/ or (switzerland or suisse or geneve or geneva or zurich or basel or bern).tw,kf,jw. | 124207 |
| 20 | Oregon/ or (oregon or portland or salem).tw,kf,jw. | 17604 |
| 21 | exp California/ or (california* or los angeles or stanford or san diego or san francisco or sacramento).tw,kf,jw. | 161788 |
| 22 | Washington/ or (washington or olympia).tw,kf,jw. | 35666 |
| 23 | Montana/ or (montana or missoula or helena).tw,kf,jw. | 5376 |
| 24 | Vermont/ or (vermont or burlington or rutland or morrisville or montpelier).tw,kf,jw. | 4267 |
| 25 | Colorado/ or (colorado or denver or aurora or lone tree).tw,kf,jw. | 27057 |
| 26 | District of Columbia/ or district of columbia.tw,kf,jw. | 12417 |
| 27 | Hawaii/ or (hawaii or honolulu or kailua or honokaa or kaneohe).tw,kf,jw. | 15584 |
| 28 | Maine/ or (maine or bangor or presque isle or blue hill or bridgton or augusta).tw,kf,jw. | 7122 |
| 29 | New Jersey/ or (new jersey or neptune or hackensack or morristown or trenton).tw,kf,jw. | 21410 |
| 30 | Australia/ or Victoria/ or (australia* or victoria or melbourne).tw,kf,jw. | 328657 |
| 31 | Luxembourg/ or (luxemb??rg or belair).tw,kf,jw. | 2633 |
| **32** | **or/16-31 [concept 3: relevant jurisdictions]** | **1421850** |
| **33** | **9 and 15 and 32** | **3300** |
