# Supplementary material for: Factors Associated With Evolution of the Use of Medical Assistance in Dying: Protocol for a Scoping Review
Source: JMIR Res Protoc. 2026 Apr 21;15:e85963. doi: 10.2196/85963 (PMC13098725; doi:10.2196/85963)
Supplement: Checklist 2 [file resprot-v15-e85963-s003.docx]

## **Multimedia Appendix 3**

**PRISMA-S Checklist (version for reporting the preliminary search strategy used for the scoping review protocol)**

| **Section/topic** | **#** | **Checklist item** | Location(s) Reported |
| --- | --- | --- | --- |
| ***Information sources and methods*** | | | |
| Database name | 1 | Name each individual database searched, stating the platform for each. | p. 8 |
| Multi-database searching | 2 | If databases were searched simultaneously on a single platform, state the name of the platform, listing all of the databases searched. | n/a |
| Study registries | 3 | List any study registries searched. | p. 8 |
| Online resources and browsing | 4 | Describe any online or print source purposefully searched or browsed (e.g., tables of contents, print conference proceedings, web sites), and how this was done. | p. 8 |
| Citation searching | 5 | Indicate whether cited references or citing references were examined, and describe any methods used for locating cited/citing references (e.g., browsing reference lists, using a citation index, setting up email alerts for references citing included studies). | p. 8 |
| Contacts | 6 | Indicate whether additional studies or data were sought by contacting authors, experts, manufacturers, or others. | Future manuscript |
| Other methods | 7 | Describe any additional information sources or search methods used. | n/a |
| ***Search strategies*** | | | |
| Full search strategies | 8 | Include the search strategies for each database and information source, copied and pasted exactly as run. | Preliminary: p. 14 (Multimedia Appendix 2) ; Full details to be reported in future manuscript |
| Limits and restrictions | 9 | Specify that no limits were used, or describe any limits or restrictions applied to a search (e.g., date or time period, language, study design) and provide justification for their use. | p. 8 |
| Search filters | 10 | Indicate whether published search filters were used (as originally designed or modified), and if so, cite the filter(s) used. | n/a |
| Prior work | 11 | Indicate when search strategies from other literature reviews were adapted or reused for a substantive part or all of the search, citing the previous review(s). | n/a |
| Updates | 12 | Report the methods used to update the search(es) (e.g., rerunning searches, email alerts). | Future manuscript |
| Dates of searches | 13 | For each search strategy, provide the date when the last search occurred. | Preliminary: p. 14 (Multimedia Appendix 2); Full details to be reported in future manuscript |
| ***Peer review*** | | | |
| Peer review | 14 | Describe any search peer review process. | Future manuscript |
| ***Managing records*** | | | |
| Total Records | 15 | Document the total number of records identified from each database and other information sources. | Future manuscript |
| Deduplication | 16 | Describe the processes and any software used to deduplicate records from multiple database searches and other information sources. | p. 8 |
|  |  |  |  |

*From:* Rethlefsen ML, Kirtley S, Waffenschmidt S, et al. PRISMA-S: an extension to the PRISMA Statement for Reporting Literature Searches in Systematic Reviews. Syst Rev. 2021;10(1):39. doi:10.1186/s13643-020-01542-z
